# Understanding More about Vaping among Asian Adolescents Aged 14-15 Years Old in New Zealand: The ASH Year 10 Snapshot Survey Analysis (2014-2022)

**DOI:** 10.1177/1179173X251359041

**Published:** 2025-07-26

**Authors:** Ko Ko, Christopher Bullen, Joanna Ting Wai Chu, Sally Frances Wong

**Affiliations:** 1 School of Population Health, University of Auckland, Auckland, New Zealand; 2 ASH – Action for Smokefree 2025, Auckland, New Zealand

**Keywords:** vaping, e-cigarettes, Asian, young people, youth, adolescents, New Zealand

## Abstract

**Background:** There has been an accelerated increase in vaping prevalence among adolescents worldwide, including in New Zealand. However, few studies have examined vaping among Asian adolescent populations in New Zealand.

**Objective:** This study aimed to investigate vaping in New Zealand adolescents over time including sources of and reasons for vaping (data available for 2021 and 2022) according to ethnicity (Asian vs Non-Asian), gender (girl vs boy vs other) and Asian major subgroupings (Chinese vs Indian vs 'Other Asian').

**Method:** We analysed the annual ASH Year 10 Snapshot Surveys of 14 and 15 year old school-children from 2014 to 2022. We used a two-sample t-test to detect the differences between two point periods and a Chi-square test to detect associations between ethnicity and sources or reasons for vaping. Binary logistic regression was used to assess vaping trends over time.

**Results:** From 2014 to 2022, vaping prevalence increased significantly in all adolescents (two-sided *P* < 0.001) while smoking prevalence decreased. However, vaping prevalence among Asian adolescents was lower and had a slower rate of growth than Non-Asian adolescents. Among Asian major subgroupings, Indian adolescents showed the highest vaping prevalence (ever vaping = 21.1%, 95% CI 18.7 – 23.7 and current vaping = 6.2%, 95% CI 4.8 – 7.8) in 2022 and, in all ethnic groupings, vaping in girls overtook that of boys. For Asian adolescents, in 2022, the most common sources of vapes was social supply (70%, *P* = 0.190) and the most common reason for vaping was experimentation (60%, *P* < 0.001).

**Conclusion:** Asian adolescents in New Zealand exhibited vaping behaviours comparable to those of their Non-Asian counterparts, with some unique patterns within subpopulations, highlighting the need for more in-depth ethnic- and gender-specific research to further examine their vaping behaviours for appropriate interventions, policies and regulations.

**What we already know**

• The prevalence of vaping among adolescents in New Zealand has increased remarkedly over recent years.
• Little is known about vaping and related behaviours among the Asian adolescent population in New Zealand.

**What this article adds**

• The prevalence of vaping among Asian adolescents in New Zealand increased year on year between 2014-2022 but with a much lower starting point and at a slower rate compared to Non-Asian adolescents.
• Vaping among adolescents identifying as Indian ethnic group showed the highest prevalence, with a unique pattern of change over time.
• In all ethnic groupings, vaping among girls has become more common than among boys recently despite the vaping prevalence in girls in 2014 being much lower.
• For Asian adolescents, the most common source of vapes was social supply: the most common reason for vaping was experimentation.
• Asian adolescents exhibited similar vaping behaviours and attitudes comparable to Non-Asian adolescents.

## Introduction

Recent international reports have highlighted an increase in vaping prevalence among adolescents worldwide, raising concerns around creating a new generation dependent on nicotine, adopting smoking-like behaviours and acting as a potential gateway to tobacco use.^1,2^ These concerns are also echoed in New Zealand – a country of 5.5 million people comprising approximately of Europeans (70%), Māori (Indigenous New Zealanders; 16%), Asian (15%), and Pacific Islanders (8%).^2,3^ While studies on adolescent vaping in New Zealand do not support the notion of a vaping epidemic, they do reveal a rapid and uneven rise in vaping rates among adolescents, with notable disparities across different ethnic groups.^2,4^ However, these studies place particular emphasis on Māori and Pacific adolescents, who have shown significantly higher rates of vaping, while offering limited insights into the experiences of Asian adoelscents who also faces distinctive challenges, including marginalization, racism, and a unique policy and legal context around vaping that may influence their risk-taking propensity.^3^ Understanding their use patterns and behaviours is also essential for informing targeted policies and programmes that could support New Zealand’s broader goal of becoming a smoke-free nation.^5^

To date, only a few surveys have focused on the vaping prevalence of adolescents in New Zealand. The Youth 2000 survey series started collecting vaping data from the adolescent age range of 13 to 18 years in 2019, so it is not possible to infer discernible trends from these few data (Long term Trends). In addition, the New Zealand Health Survey (NZHS) also collects the vaping prevalence of people aged 15 years and above, annually, but the sample size of adolescents aged 15 to 17 years is small, making the estimates less precise than much larger surveys.^6^ The biennial Youth Insights Surveys collecting information about tobacco proximal risk factors for vaping and covering approximately 2800 students aged 14-15 years since 2006 was discontinued in 2018.^6^ On the other hand, the ASH Year 10 Snapshot Survey (covering approximately 250 000 Year 10 students) has provided a comprehensive overview of smoking for many years and in recent years has included questions about vaping, offering valuable insights into the growing trend of adolescent vaping and related behaviours in New Zealand.^7^ All New Zealand adolescent data sources on tobacco and vaping are shown in Appendix A.

Drawing on these annual ASH Year 10 surveys, this study aims to investigate vaping specifically on Asian adolescents in New Zealand. It will focus on ever and current vaping to compare the changes in rates among Asian adolescents in contrast to that of Non-Asian over time (from 2014 to 2022). The study will investigate their use patterns over time and will examine sources of vapes and reasons for vaping together with other vaping behavioural and attitudinal questions in the survey (available for 2021 and 2022) according to ethnicity, gender and Asian subgroupings, particularly Chinese and Indian adolescents who constitute a majority of the Asian population in New Zealand.

## Method

### Data Sources and Study Population

The Year 10 Snapshot survey, a census style survey, is one of the largest ongoing youth smoking surveys in the world. It began in 1992 and has included questions about smoking since 1999 and vaping since 2014.^7^ Each year, all New Zealand schools with Year 10 students (other than distant education) are invited to participate. About 25 000 - 30 000 students (around 40% of New Zealand Year 10 adolescents aged 14 and 15 years) participate each year. The total sample size for this study is 213 483 (8 years of data from 2014 to 2022 except 2020). School principals provide written consent on behalf of participating schools, and students are given the option to opt out anytime. Details of participating schools and the answers given by students are kept confidential.

The survey uses robust and validated measures, and is conducted to a high methodological standard that has been subject to peer review and ethics approval. The survey is funded by Health New Zealand / Te Whatu Ora, as part of the New Zealand Youth Tobacco Monitor (NZYTM). The surveys have 22-24 questions and the survey questions with response options for each year are provided in the ASH website.^7^ Questions include age, gender, self-identified ethnicity (students can select more than one ethnicity) and school decile (an area-based socioeconomic measure used to allocate more resources to schools in the lowest deciles). Gender options are male and female, with a third option, gender diverse included since 2019. Smoking and vaping questions related to use patterns ask about ever use and frequency of use. For vaping, since 2021, more questions were added to ask how adolescents are obtaining vapes in the past 30 days, their main reasons for vaping, and other vaping behavioral and attitudinal questions, such as types of vape used, vaping with nicotine, whether they smoked first or vaped first, plan to try vapes soon and willingness to vape if their best friends offer one to them. Completed questions are collated by schools and are returned by courier for checking to ASH (a non-governmental organisation focused on advancing the Smokefree New Zealand vision). Spoiled and/or incomplete questionaires are removed to reduce data entry error.

The Multiregional Health and Disability Ethics Committee approved all ASH survey waves (MEC/07/10/141).

### Data Analysis

We used the StatsNZ standard to prioritize ‘ethnic group’ and combined Māori, Pacific, and European adolescent populations into a single grouping called ‘Non-Asian’, and all the Asian ethnic groups into a single ‘Asian’ grouping.^2^ While the combination of Non-Asian groups as a single grouping masks important ethnic differences between and within those groups, it makes the primary comparison with Asians much clearer. Among many Asian ethnic groups, we created 3 major subgroupings: Chinese (those who responded ‘Yes’ to the question on ethnic identity as “ethnic chinese only”), Indian (those who responded ‘Yes’ to “ethnic indian only”), and ‘Other Asian’ (those who responded ‘Yes’ to both *ethnic chinese* and *ethnic indian* as well as other Asian ethnicities such as Japanese, Korean, etc.). We merged ‘Gender diverse’ and ‘Another gender’ into a new gender category called ‘other’.

Next, we summarised the demographic distributions in number and proportion for the overall sample by year, by ethnicity (Asian, Non-Asian), by Asian major subgroupings (Chinese, Indian, ‘Other Asian’) and by gender (girl, boy, other), and calculated the prevalence of vaping and smoking in number and proportions with 95% confident intervals from 2014 to 2022 to enable comparisons of prevalence. Ever use means ‘ever tried an e-cigarette or cigarette once in their lifetime’ and current use means ‘used in the past 30 days, either daily or weekly or monthly’. For vaping, the current use data was available starting from 2015. We excluded ‘Less than monthly’ answers in both vaping and smoking because they are more likely to reflect experimentation.

Three main sources of vapes and 5 main reasons for vaping were categorized depending on availability of data in the survey. Sources of vapes were grouped as: (1) Physical or online retailers (vape shop, a supermarket, or diary, or petrol station or convenient store or other shop, and online); (2) Social supply (bought or received or took without asking from friends or person with same age, or some or brother or sister, or parent or caregiver; (3) Other sources. Reasons of vaping were grouped as: (1) Experimentation; (2) Pleasure (like the flavours, other people use, enjoy, and looking cool; (3) Cutting down or reducing smoking (instead of smoking, quit smoking, and cut down smoking cigarettes; (4) Affordability and accessibility (cheaper than smoking and easier to get than cigarettes; (5) Other reasons. For vaping behavioural and attitudinal questions (asked only in the 2021 and 2022 surveys), we combined ‘Definitely not’ and ‘Probably not’ answers into single ‘No’ responses while ‘Probably yes’ and ‘Definitely yes’ answers were combined into single ‘Yes’ responses, followed by calculating them in number and proportions only.

Missing values were managed on a variable-by-variable basis. As the pattern of missing data was found to be random across age groups, gender and ethnicities, we used a pairwise deletion method to maintain sample size and preserve statistical power.^8^ Regarding demographic data and ever use patterns of vaping and smoking, there is no missing data as only complete information related to them were included for data entry. For other variables, missing data was 0.5% for frequency of smoking, 1.2% for frequency of vaping, 3.5% for sources of vapes and 4.6% for reasons for vaping. ‘Don’t know’ response was only asked for reasons for vaping and was 2.1%. We excluded both missing data and ‘Don’t know’ responses from the numerator and denominator during analysis.

### Statistical Analysis

We used Stata^TM^ software to calculate standard errors (SEs) and proportions.^9^ A Chi-square test was used to detect associations between ethnicity and source of adolescent vaping and between ethnicity and major reasons for adolescent vaping. We also evaluated if there was any association among Asian major subgroupings. For gender, we were unable to analyse associations for Asian major subgroupings due to their small sample sizes. We used a two-sample t-test to detect if differences in the variables of interest between 2 time periods were significant. Finally, using IBM SPSS Statistics (Version 25) we used a binary logistic regression test for a linear trend in the prevalence of ever and current vaping by Asian and Asian major subgroupings.^10,11^

## Results

Table 1 shows the results of the main analyses of categories of vaping and smoking.

**Table 1.** Number and Proportion (95% CI) of New Zealand Adolescents Self-Reported E-cigarette and Cigarette Use by Ethnicity, by Asian Major Subgroupings, by Ethnicity and by Gender From 2014 to 2022

Year	Total sample (n)	Ever use		Current use	
		*E-cigarette	Cigarette	*E-cigarette	Cigarette
Ethnicity					
Asian					
2014**	3797 (12.5%)	405 (10.7%) (9.7 – 11.7)	329 (8.7%)	NA	275 (7.2%)
2015	2274 (10.7%)	204 (9.0%) (7.8 – 10.2)	183 (8.0%)	36 (1.6%) (1.1 – 2.2)	158 (6.9%)
2016	2882 (11.7%)	312 (10.8%) (9.7 – 12.0)	217 (7.5%)	53 (1.8%) (1.4 – 2.4)	169 (5.9%)
2017	3306 (12.4%)	389 (11.8%) (10.7 – 12.9)	229 (6.9%)	84 (2.5%) (2.0 – 3.1)	195 (5.9%)
2018	4044 (14.2%)	563 (13.9%) (12.9 – 15.0)	243 (6.0%)	94 (2.3%) (1.9 – 2.8)	210 (5.2%)
2019	3954 (14.6%)	624 (15.8%) (14.7 – 17.0)	235 (5.9%)	137 (3.5%) (2.9 – 4.1)	190 (4.8%)
2021	3729 (14.2%)	708 (19.0%) (17.7 – 20.3)	223 (6.0%)	215 (5.8%) (5.0 – 6.6)	176 (4.7%)
2022**	4228 (14.7%)	694 (16.4%) (15.3 – 17.6)	189 (4.5%)	192 (4.5%) (3.9 – 5.2)	160 (3.8%)
Non-Asian					
2014**	26 646 (87.5%)	5932 (22.3%) (21.8 – 22.8)	6616 (25%)	NA	5326 (20.2%)
2015	18 960 (89.3%)	4683 (24.7%) (24.1 – 25.3)	4290 (23%)	694 (3.7%) (3.4 – 3.9)	3632 (19.2%)
2016	21 674 (88.3%)	5837 (26.9%) (26.3 – 27.5)	4807 (22%)	1008 (4.7%) (4.4 – 4.9)	3559 (16.4%)
2017	23 414 (87.6%)	7387 (31.5%) (31.0 – 32.1)	4558 (19%)	1650 (7.0%) (6.7 – 7.4)	3584 (15.3%)
2018	24 389 (85.8%)	8917 (36.6%) (36.0 – 37.2)	5112 (20%)	1948 (8.0%) (7.6 – 8.3)	3923 (16.1%)
2019	23 129 (85.4%)	9469 (40.9%) (40.3 – 41.6)	5053 (21%)	3048 (13.2%) (12.7 – 13.6)	3875 (16.8%)
2021	22 449 (85.7%)	10 464 (46.6%) (46.0 – 47.3)	4368 (19%)	5036 (22.4%) (21.9 – 23.0)	3131 (13.9%)
2022**	24 604 (85.3%)	10 879 (44.2%) (43.6 – 44.8)	3906 (16%)	5006 (20.3%) (19.8 – 20.9)	2846 (11.6%)
Asian major subgroupings					
*Chinese*					
2014**	1143 (30.1%)	93 (8.1%) (6.6 – 9.9)	78 (6.8%)	NA	70 (6.1%)
2015	734 (32.3%)	49 (6.7%) (5.0 – 8.7)	46 (6.3%)	9 (1.2%) (0.6 – 2.3)	40 (5.4%)
2016	839 (29.1%)	63 (7.5%) (5.8 – 9.5)	51 (6.1%)	12 (1.4%) (0.7 – 2.5)	41 (4.9%)
2017	1074 (32.5%)	92 (8.6%) (7.0 – 10.4)	64 (6.0%)	17 (1.6%) (0.9 – 2.5)	57 (5.3%)
2018	1206 (29.8%)	123 (10.2%) (8.5 – 12.0)	55 (4.6%)	17 (1.4%) (0.8 – 2.2)	48 (4.0%)
2019	1211 (30.6%)	135 (11.1%) (9.4 – 13.1)	50 (4.1%)	33 (2.7%) (1.9 – 3.8)	44 (3.6%)
2021	1105 (29.6%)	136 (12.3%) (10.4 – 14.4)	48 (4.3%)	41 (3.7%) (2.7 – 5.0)	40 (3.6%)
2022**	1497 (35.4%)	160 (10.7%) (9.2 – 12.4)	51 (3.4%)	46 (3.1%) (2.3 – 4.1)	44 (2.9%)
*Indian*					
2014**	1012 (26.7%)	132 (13.0%) (11.0 – 15.3)	91 (9.0%)	NA	74 (7.3%)
2015	577 (25.4%)	54 (9.4%) (7.1 – 12.0)	53 (9.2%)	11 (1.9%) (1.0 – 3.4)	43 (7.5%)
2016	650 (22.6%)	83 (12.8%) (10.3 – 15.6)	51 (7.8%)	19 (2.9%) (1.8 – 4.5)	39 (6.0%)
2017	724 (21.9%)	111 (15.3%) (12.8 – 18.2)	63 (8.7%)	28 (3.9%) (2.6 – 5.5)	52 (7.2%)
2018	939 (23.2%)	146 (15.5%) (13.3 – 18.0)	50 (5.3%)	33 (3.5%) (2.4 – 4.9)	47 (5.0%)
2019	878 (22.2%)	163 (18.6%) (16.0 – 21.3)	52 (5.9%)	47 (5.4%) (4.0 – 7.0)	40 (4.6%)
2021	817 (21.9%)	153 (18.7%) (16.1 – 21.6)	40 (4.8%)	48 (5.9%) (4.4 – 7.7)	29 (3.5%)
2022**	1083 (25.6%)	229 (21.1%) (18.7 – 23.7)	50 (4.6%)	67 (6.2%) (4.8 – 7.8)	39 (3.6%)
*‘Other Asian’*					
2014**	1642 (42.2%)	180 (11.0%) (9.5 – 12.6)	160 (9.7%)	NA	131 (8.0%)
2015	963 (42.3%)	101 (10.5%) (8.6 – `1.6)	84 (8.7%)	16 (1.7%) (1.0 – 2.7)	75 (7.8%)
2016	1393 (48.3%)	166 (11.9%) (10.3 – 13.7)	115 (8.3%)	22 (1.6%) (1.0 – 2.4)	89 (6.4%)
2017	1508 (45.6%)	186 (12.3%) (10.7 – 14.1)	102 (6.8%)	39 (2.6%) (1.8 – 3.5)	86 (5.7%)
2018	1899 (47.0%)	294 (15.5%) (13.9 – 17.2)	138 (7.3%)	44 (2.3%) (1.7 – 3.1)	115 (6.1%)
2019	1865 (47.2%)	326 (17.5%) (15.8 – 19.3)	133 (7.1%)	57 (3.1%) (2.3 – 3.9)	106 (5.7%)
2021	1807 (48.5%)	419 (23.2%) (21.3 – 25.2)	135 (7.5%)	126 (7.0%) (5.8 – 8.2)	107 (5.9%)
2022**	1648 (39.0%)	305 (18.5%) (16.7 – 20.5)	88 (5.3%)	79 (4.8%) (3.8 – 5.9)	77 (4.7%)
Gender					
Asian					
*Girl*					
2014**	1933 (50.9%)	163 (8.4%) (7.2 – 9.8)	148 (7.7%)	NA	121 (6.3%)
2015	1122 (49.3%)	73 (6.5%) (5.2 – 8.1)	70 (6.2%)	11 (1.0%) (0.5 -1.7)	59 (5.3%)
2016	1591 (55.2%)	114 (7.2%) (5.9 – 8.5)	96 (6.0%)	17 (1.1%) (0.6 – 1.7)	77 (4.8%)
2017	1767 (53.4%)	154 (8.7%) (7.4 – 10.1)	105 (5.9%)	35 (2.0%) (1.4 – 2.7)	91 (5.1%)
2018	2174 (53.8%)	251 (11.5%) (10.2 – 13.0)	109 (5.0%)	35 (1.6%) (1.1 – 2.2)	94 (4.3%)
2019	1978 (50.0%)	231 (11.7%) (10.3 – 13.2)	95 (4.8%)	45 (2.3%) (1.7 – 3.0)	78 (3.9%)
2021	1864 (50.0%)	347 (18.6%) (16.9 – 20.5)	101 (5.4%)	129 (6.9%) (5.8 – 8.2)	74 (4.0%)
2022**	2129 (50.4%)	347 (16.3%) (14.8 – 18.0)	82 (3.9%)	109 (5.1%) (4.2 – 6.1)	67 (3.1%)
Boy					
2014**	1864 (49.1%)	242 (13.0%) (11.5 – 14.6)	181 (9.7%)	NA	154 (8.3%)
2015	1152 (50.7%)	131 (11.4%) (9.6 – 13.3)	113 (9.8%)	25 (2.2%) (1.4 – 3.2)	99 (8.6%)
2016	1291 (44.8%)	198 (15.3%) (13.4 – 17.4)	121 (9.4%)	36 (2.8%) (2.0 – 3.8)	92 (7.1%)
2017	1539 (46.6%)	235 (15.3%) (13.5 – 17.2)	124 (8.1%)	49 (3.2%) (2.4 – 4.2)	104 (6.8%)
2018	1870 (46.2%)	312 (16.7%) (15.0 – 18.5)	134 (7.2%)	59 (3.2%) (2.4 – 4.1)	116 (6.2%)
2019	1915 (48.4%)	363 (19.0%) (17.2 – 20.8)	114 (6.0%)	76 (4.0%) (3.1 – 4.9)	92 (4.8%)
2021	1803 (48.4%)	343 (19.0%) (17.2 – 20.9)	116 (6.4%)	82 (4.5%) (3.6 – 5.6)	96 (5.3%)
2022**	2029 (48.0%)	329 (16.2%) (14.6 – 17.9)	96 (4.7%)	77 (3.8%) (3.0 – 4.7)	85 (4.2%)
Other					
2019**	61 (1.5%)	30 (49.2%) (36.1 – 62.3)	26 (42.6%)	16 (26.2%) (15.8 – 39.1)	20 (32.8%)
2021	62 (1.7%)	18 (29.0%) (18.2 – 41.9)	6 (9.7%)	4 (6.5%) (1.8 – 15.7)	6 (9.7%)
2022**	70 (1.7%)	18 (25.7%) (16.0 – 37.6)	11 (15.7%)	6 (8.6%) (3.2 – 17.7)	8 (11.4%)
Non-Asian					
Girl					
2014**	13 770 (51.7%)	2735 (19.9%) (19.2 – 20.5)	3582 (26.0%)	NA	2810 (20.4%)
2015	9551 (50.4%)	2001 (21.0%) (20.1 – 21.8)	2253 (23.6%)	268 (2.8%) (2.5 – 3.2)	1906 (20.0%)
2016	11 405 (52.6%)	2583 (22.6%) (21.9 – 23.4)	2505 (22.0%)	403 (3.5%) (3.2 – 3.9)	1802 (15.8%)
2017	12 043 (51.4%)	3297 (27.4%) (26.6 – 28.2)	2419 (20.1%)	667 (5.5%) (5.1 – 6.0)	1837 (15.3%)
2018	12 813 (52.5%)	4173 (32.6%) (31.8 – 33.4)	2716 (21.2%)	890 (6.9%) (6.5 – 7.4)	2055 (16.0%)
2019	11 657 (50.4%)	4329 (37.1%) (36.3 – 38.0)	2564 (22.0%)	1397 (12.0%) (11.4 – 12.6)	1912 (16.4%)
2021	11 199 (49.9%)	5392 (48.1%) (47.2 – 49.1)	2296 (20.5%)	2843 (25.4%) (24.6 – 26.2)	1598 (14.3%)
2022**	12 150 (49.4%)	5709 (47.0%) (46.1 – 47.9)	2041 (16.8%)	2852 (23.5%) (22.7 – 24.2)	1419 (11.7%)
Boy					
2014**	12 876 (48.3%)	3197 (24.8%) (24.1 – 25.6)	3034 (23.6%)	NA	2975 (19.5%)
2015	9409 (49.6%)	2682 (28.5%) (27.6 – 29.4)	2037 (21.6%)	426 (4.5%) (4.1 – 5.0)	2004 (18.3%)
2016	10 269 (47.4%)	3254 (31.7%) (30.8 – 32.6)	2302 (22.4%)	605 (5.9%) (5.4 – 6.4)	2080 (17.1%)
2017	11 371 (48.6%)	4090 (36.0%) (35.1 – 36.9)	2139 (18.8%)	983 (8.6%) (8.1 – 9.2)	2110 (15.4%)
2018	11 576 (47.5%)	4744 (41.0%) (40.1 – 41.9)	2396 (20.7%)	1058 (9.1%) (8.6 – 9.7)	2353 (16.1%)
2019	11 087 (47.9%)	4912 (44.3%) (43.4 – 45.2)	2336 (21.1%)	1541 (13.9%) (13.3 – 14.6)	2305 (16.6%)
2021	10 773 (48.0%)	4869 (45.2%) (44.3 – 46.1)	1954 (18.1%)	2106 (19.5%) (18.8 – 20.3)	1904 (13.4%)
2022**	11 858 (48.2%)	4930 (41.6%) (40.7 – 42.5)	1744 (14.7%)	2047 (17.3%) (16.6 – 18.0)	1705 (11.2%)
Other					
2019**	385 (1.7%)	228 (59.2%) (54.1 – 64.2)	153 (39.7%)	110 (28.6%) (24.1 – 33.4)	119 (30.9%)
2021	477 (2.1%)	203 (42.6%) (38.1 – 47.1)	118 (24.7%)	87 (18.2%) (14.9 – 22.0)	92 (19.3%)
2022**	596 (2.4%)	240 (40.3%) (36.3 – 44.3)	121 (20.3%)	107 (18.0%) (15.0 – 21.3)	94 (15.8%)

NA = not available, Ever use = ever tried at least once, Current use of e-cigarettes (daily, weekly, and monthly), Current use of cigarettes (experimental, daily, weekly, and monthly), * = binary logistic regression was performed over time (Two sided *P* < 0.000 in both ever and current cigarette use), ** = Two sample T-test was performed (*P* < 0.05 in both ever and current cigarette use irrespective of ethnicity and gender).

Remark* For cigarette use, only numbers and proportions were calculated without 95% CI.

The sample size of the ASH Year 10 Snapshot Survey ranged from 30 443 in 2014 to 28 832 in 2022. The proportion of participants in each sample who identified as Asian ethnicity varied from 12.5% in 2014 to 14.7% in 2022 with an average of 13.1% of the total study population each year. Among the Asian group, the proportion of participants who identified as Indian ethnicity declined slightly from 26.7% in 2014 to 25.6% in 2022, Chinese ethnicity rose from 30.1% in 2014 to 35.4% in 2022, and ‘Other Asian’ declined from 42.2% in 2014 to 39.0% in 2022. Gender distribution for the Asian population including Asian subgroupings showed little difference with that of Non-Asian groups: girls were around 50% and boys around 48% of the sample. Those categorised as ‘other’ gender comprised just 2% of the study population since it started being collected. (See Table 1)

### Prevalence of A dolescent Vaping in New Zealand from 2014 to 2022 (by ethnicity and by gender)

**Figure 1.**
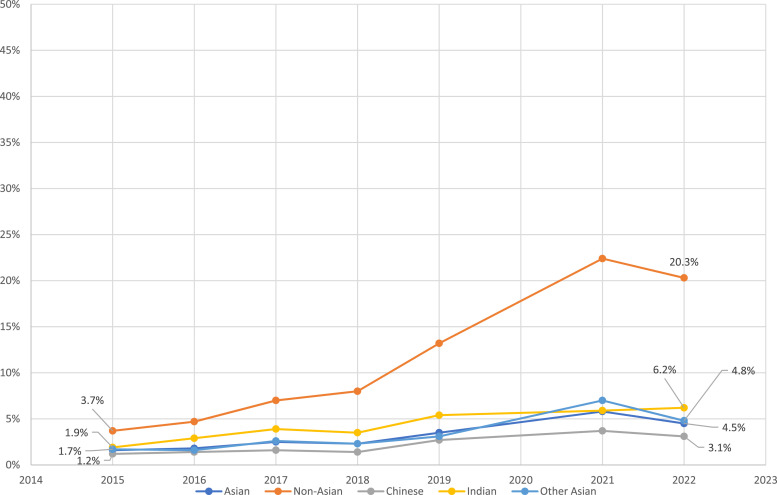
Prevalence of Current Vaping by Ethnicity and by Asian Major Subgroupings, New Zealand Adolescents Aged 14 and 15, 2015-2022

Table 1 shows that the prevalence of Asian adolescents who reported ever vaping and current vaping increased significantly (both, *P* < 0.05) from 10.7% in 2014 to 16.4% in 2022 and from 1.6% in 2015 to 4.5% in 2022 and those of Non-Asian adolescents showed a statistically significant increase (both *P* < 0.05) in prevalence of ever and current vaping, from 22.3% in 2014 to 44.2% in 2022 and 3.7% in 2015 to 20.3% in 2022, respectively. (See Figure 1) This pattern was the same for gender analysis among ethnicity and Asian major subgroupings.

**Figure 2.**
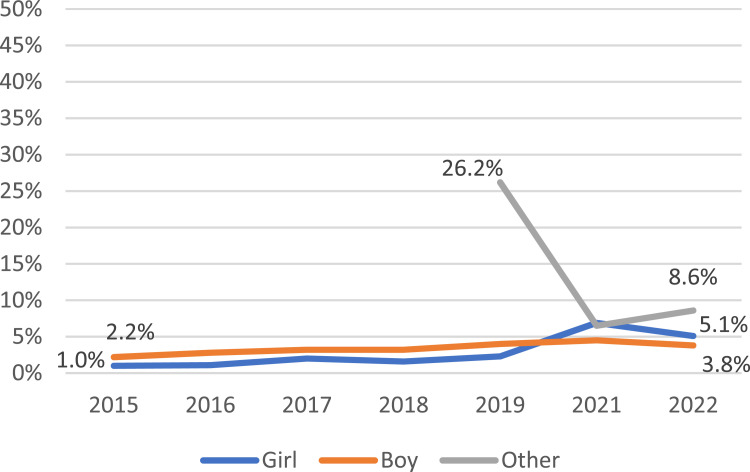
Prevalence of Current Vaping by Gender, New Zealand Asian Adolescents Aged 14 and 15, 2015 - 2022

**Figure 3.**
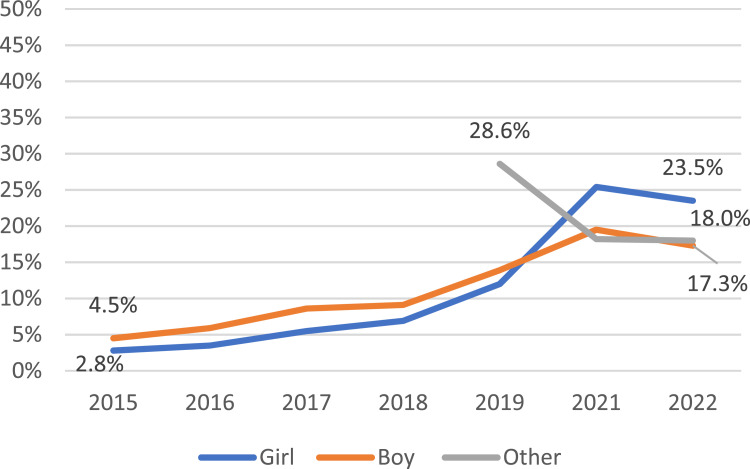
Prevalence of Current Vaping by Gender, New Zealand Non-Asian Adolescents Aged 14 and 15, 2015 - 2022

The prevalence of ever vaping by Asian girls almost doubled from 8.4% in 2014 to 16.3% in 2022 while the prevalence of current vaping increased significantly from a low starting point of 1.0% in 2015 to 5.1% in 2022. For Asian boys, changes were less dramatic: the prevalence of ever vaping increased slightly from 13.0% in 2014 to 16.2% in 2022 and current vaping from 2.2% in 2015 to 3.8% in 2022. (See Figure 2) In contrast, there were major shifts in vaping in the Non-Asian group: the baseline of vaping was far higher than in Asian adolescents, the prevalence of ever and current vaping more than doubled from 19.9% in 2014 to 47.0% in 2022 and jumped from 2.8% in 2015 to 23.5% in 2022 for girls, almost doubled from 24.8% in 2014 to 41.6% in 2022 and more than tripled from 4.5% in 2015 to 17.3% in 2022 for boys, respectively. (See Figure 3) For those identifying as ‘other’ gender, ever and current vaping by Asian decreased, from 49.2% in 2019 to 25.7% in 2022 and from 26.2% in 2019 to 8.6% in 2022, and in Non-Asians from 59.2% in 2019 to 40.3% in 2022 and from 28.6% in 2019 to 18.0% in 2022, respectively. (See Figures 2, 3)

### Prevalence of Adolescent Vaping in New Zealand from 2014 to 2022 (by Asian Major Subgroupings)

The prevalence of ever vaping by Chinese, Indian and ‘Other Asian’ adolescents showed an increase from 8.1% in 2014 to 10.7% in 2022, from 13.0% in 2014 to 21.1% in 2022, and from 11.0% in 2014 to 18.5% in 2022, respectively. As with the prevalence of ever vaping, from 2015 to 2022, the prevalence of current vaping by Chinese increased from 1.2% to 3.1%, Indian from 1.9% to 6.2% and the ‘Other Asian’ group from 1.7% to 4.8%. (See Table 1)

The prevalence of ever and current smoking, comparing Asian including Asian major subgroupings and Non-Asian adolescents are shown in Appendix B. These data are included because, while the focus is on vaping, it is important to understand changes in vaping in relation to trends in smoking in the same population groups over the same period.

### Sources of and Reasons for Vaping

Sources of and reasons for vaping, and responses to questions about vaping behaviour and attitudes are shown in Table 2. In all groupings, including Asian major subgroupings, social supply was the most common source of vapes (70%), followed by ‘other sources' with around 15-20 % in both years. Physical or online retailers were relatively uncommon sources except in Chinese adolescents, where they became the second most common source of vaping in 2022. Experimentation was the most common reason provided for vaping, with around 60% for Asians (including each major subgrouping) and around 45% for Non-Asians. Pleasure was the next most common reason provided, by around 30% for Asians (including each major subgrouping) and around 40% for Non-Asians, in both 2021 and 2022. The remaining three reasons were relatively rare answers, with each category contributing just under 10% in both years.

**Table 2.** Numbers and Proportions of Sources of Vaping, Reasons for Vaping, and Answers to Behaviour and Attitude Questions by New Zealand Adolescents, by Ethnicity, by Asian Major Subgroupings, 2021-2022

Category	Asian		Non-Asian		Chinese		Indian		‘Other Asian’	
	2021	2022	2021	2022	2021	2022	2021	2022	2021	2022
Sources of vaping										
Physical or online retailers^a^	27 (7.2%) (4.8 – 10.2)	30 (9.3%) (6.4 – 13.1)	517 (7.6%) (7.0 – 8.3)	725 (11.3%) (10.6 – 12.1)	8 (12.5%) (5.6 – 23.2)	12 (19.7%) (10.6 – 31.8)	7 (7.9%) (3.2 – 15.5)	9 (7.1%) (3.3 – 13.0)	12 (5.4%) (2.8 – 9.2)	9 (6.8%) (3.1 – 12.5)
*Social supply^b^	294 (78.0%) (73.5 – 82.1)	232 (72.3%) (67.0 – 77.1)	5073 (74.9%) (73.9 – 76.0)	4398 (68.8%) (67.7 – 69.9)	46 (71.9%) (59.2 – 82.4)	39 (63.9%) (50.6 – 75.8)	69 (77.5%) (67.4 – 85.7)	91 (71.7%) (63.0 – 79.3)	179 (79.9%) (74.1 – 85.0)	102 (76.7%) (68.6 – 83.6)
Other sources^c^	56 (14.9%) (11.4 – 18.9)	59 (18.4%) (14.3 – 23.1)	1180 (17.4%) (16.5 – 18.4)	1269 (19.9%) (18.9 – 20.9)	10 (15.6%) (7.8 – 26.9)	10 (16.4%) (8.2 – 28.1)	13 (14.6%) (8.0 – 23.7)	27 (21.3%) (14.5 – 29.4)	33 (14.7%) (10.3 – 20.1)	22 (16.5%) (10.7 – 24.0)
Reasons for vaping										
*Experimentation^d^	259 (59.8%) (55.1 – 64.5)	235 (63.0%) (57.9 – 67.9)	2938 (44.5%) (43.3 – 45.7)	2963 (45.5%) (44.3 – 46.7)	41 (51.9%) (40.3 – 63.3)	51 (60.0%) (48.8 – 70.5)	67 (59.3%) (49.6 – 68.4)	85 (57.4%) (49.0 – 65.5)	151 (53.7%) (47.7 – 59.7)	99 (57.2%) (49.5 – 64.7)
*Pleasure^e^	150 (34.6%) (30.2 – 39.3)	115 (30.8%) (26.2 – 35.8)	2644 (40.1%) (38.9 – 41.3)	2601 (40.0%) (38.8 – 41.2)	28 (35.4%) (25.0 – 47.0)	24 (28.2%) (19.0 – 39.0)	35 (31.0%) (22.6 – 40.4)	45 (30.4%) (23.1 – 38.5)	87 (31.0%) (25.6 – 36.7)	46 (26.6%) (20.2 – 33.8)
Cutting down or reducing smoking^f^	14 (3.2%) (1.8 – 5.4)	12 (3.2%) (1.7 – 5.6)	425 (6.4%) (5.9 – 7.1)	273 (4.2%) (3.7 – 4.7)	1 (1.3%) (0.0 – 6.9)	3 (3.5%) (0.7 – 10.0)	3 (2.7%) (0.6 – 7.6)	6 (4.1%) (1.5 – 8.6)	10 (3.6%) (1.7 – 6.4)	3 (1.7%) (0.4 – 5.0)
Affordability and accessibility^g^	2 (0.5%) (0.1 – 1.7)	3 (0.8%) (0.2 – 2.3)	79 (1.2%) (0.9 – 1.5)	66 (1.0%) (0.8 – 1.3)	0	1 (1.2%) (0.0 – 6.4)	1 (0.9%) (0.0 – 4.8)	1 (0.7%) (0.0 – 3.7)	1 (0.4%) (0.0 – 2.0)	1 (0.6%) (0.0 – 3.2)
Other reasons^h^	8 (1.8%) (1.0 – 3.6)	8 (2.1%) (0.9 – 4.2)	513 (7.8%) (7.1 – 8.4)	606 (9.3%) (8.6 – 10.0)	9 (11.4%) (5.3 – 20.5)	6 (7.1%) (2.6 – 14.7)	7 (6.2%) (2.5 – 12.3)	11 (7.4%) (3.8 – 12.9)	32 (11.4%) (7.9 – 15.7)	24 (13.9%) (9.1 – 20.0)
Vaping behavioural and attitudinal questions										
Type of e-cigarettes used										
Disposable e-cigarettes	55 (17.5%)	124 (49.6%)	857 (15.2%)	2602 (47.4%)	14 (23.0%)	30 (54.5%)	14 (18.7%)	47 (51.6%)	27 (15.2%)	47 (45.2%)
Rechargeable e-cigarettes with replaceable prefilled catridges	91 (29.0%)	52 (20.8%)	1436 (25.4%)	1003 (18.3%)	12 (19.7%)	8 (14.5%)	26 (34.7%)	17 (18.7%)	53 (29.8%)	27 (26.0%)
Rechargeable e-cigarettes with a tank or reservoir filled with liquid	168 (53.5%)	74 (29.6%)	3363 (59.5%)	1882 (34.3%)	35 (57.4%)	17 (30.9%)	35 (46.7%)	27 (29.7%)	98 (55.1%)	30 (34.3%)
Number and proportion of New Zealand adolescents who answered either ‘smoked first’ or ‘Vaped first’										
Smoked first	102 (18.0%)	68 (13.2%)	1862 (21.7%)	1506 (17.7%)	24 (21.2%)	19 (16.7%)	20 (6.8%)	18 (8.2%)	58 (36.3%)	31 (17.1%)
Vaped first	465 (82.0%)	446 (86.8%)	6704 (78.3%)	6984 (82.3%)	89 (78.8%)	95 (83.3%)	274 (93.2%)	201 (91.8%)	102 (63.8%)	150 (82.9%)
Number and proportion of New Zealand adolescents who answered ‘Vaping with or without nicotine’										
Without nicotine	101 (24.0%)	45 (14.0%)	1237 (17.7%)	752 (11.4%)	16 (21.6%)	9 (12.7%)	26 (28.6%)	19 (16.8%)	59 (23.0%)	17 (12.4%)
With nicotine	320 (76.0%)	276 (85.9%)	5763 (82.3%)	5871 (88.6%)	58 (78.4%)	62 (87.3%)	65 (71.4%)	94 (83.2%)	197 (77.0%)	120 (87.6%)
Number and proportion of New Zealand adolescents who answered ‘No’ or ‘Yes’ to trying vapes soon										
No	3280 (88.4%)	3864 (91.8%)	15 358 (69.4%)	17 899 (73.7%)	1017 (92.2%)	1409 (94.5%)	707 (86.7%)	964 (89.5%)	1556 (86.7%)	1491 (90.9%)
Yes	432 (11.6%)	344 (8.2%)	6770 (30.6%)	6398 (26.3%)	86 (7.8%)	82 (5.5%)	108 (13.3%)	113 (10.5%)	238 (13.3%)	140 (9.1%)
Number and proportion of New Zealand adolescents who answered ‘No’ or ‘Yes’ to vaping if best friend offered										
No	3191 (86.0%)	3799 (90.2%)	14 533 (65.5%)	17 052 (70.0%)	994 (90.1%)	1390 (93.3%)	698 (85.9%)	944 (87.5%)	1499 (83.6%)	1465 (89.2%)
Yes	519 (14.0%)	413 (9.8%)	7645 (34.5%)	7306 (30.0%)	109 (9.9%)	100 (6.7%)	115 (14.1%)	135 (12.5%)	295 (16.4%)	178 (10.8%)

^a^Physical or online retailers includes vape shop, a supermarket, or dairy or petrol station or convenient store or other shop and online.

^b^Social supply includes bought or received or took without asking from friends or person of same age or someone or brother or sister or parent or caregiver.

^c^Other sources = not specified.

^d^Experimentation includes trial.

^e^Pleasure includes like the flavours, other people use, enjoyment, looking cool.

^f^Cutting down or reducing smoking includes instead of smoking, quit smoking, cut down smoking cigarettes.

^g^Affordability and accessibility includes cheaper than smoking, easier to get than cigarettes.

^h^Other reasons = not specified.

“Remark” For answers to vaping behavioural and attitudinal questions, only numbers and proportions of answers (without 95% CI) was calculated.* = Chi-square test was performed to identify association among ethnicity and among Asian major subgroupings. Significant association was found only between ethnicity and vaping for experimentation and pleasure reasons.

There was no significant association between ethnicity and social supply (x^2^ (1, N = 7147) = 1.77, *P* = 0.183 in 2021 and x^2^ (1, N = 6713) = 1.71, *P* = 0.190 in 2022) and between social supply and Asian major subgroupings. However, there was a significant association between ethnicity and vaping for experimentation (x^2^ (1, N = 7032) = 38.33, *P* < 0.001 in 2021 and x^2^ (1, N = 6882) = 43.33, *P* < 0.001 in 2022) and for pleasure (x^2^ (1, N = 7032) = 4.99, *P* < 0.001 in 2021 and x^2^ (1, N = 6882) = 12.30, *P* < 0.001 in 2022). No significant association was seen between any reasons for vaping and Asian major subgroupings.

### Trends in Vaping from 2014 to 2022

Binary logistic regression for linear trends of vaping in Asian (including Asian major subgroupings) and Non-Asian adolescents from 2014 to 2022 found a statistically significant trend (both ever and current vaping) over time in these groups (two-sided *P* < 0.001 for all groups).

### Vaping Behavioural and Attitudinal Questions and Answers by New Zealand Adolescents at a Glance

Rechargeable vapes with a tank or reservoir filled with liquid were the most popular type in 2021 used by over 50% of both Asian and Non-Asian adolescents, but in 2022, disposable vapes became more popular, used by 49.6% of Asian adolescents and 47.4% of Non-Asian adolescents who had vaped. Asian adolescents (85%) were more likely to answer ‘Vaped first’ than Non-Asians (80%). Over 80% of both Asian and Non-Asian adolescents answered ‘Vaping with nicotine’. Non-Asian adolescents (30%) were more likely to answer ‘Yes’ to the question ‘Trying vapes soon’ than Asian (10%). The proportion of Asian adolescents who showed willingness to vape if their best friends offered was much lower compared to Non-Asian adolescents, with around 12% for Asian, and around 30% for Non-Asian. (See table 2)

## Discussion

From 2014 to 2022, the prevalence of vaping among both Asian and Non-Asian adolescents aged 14 to 15 years increased significantly while the prevalence of smoking decreased. But the prevalence among Asian adolescents was consistently lower than that of Non-Asian adolescents. Among Asian major subgroupings, Indian adolescents showed the highest increase in both ever and current vaping. The prevalence among girls showed a higher increase compared to that of boys in all ethnic groupings. Experimentation was found to be a major factor associated with vaping in Asian adolescents.

Like other studies, this study shows a significant increase in the prevalence of vaping among New Zealand adolescents over time.^2,4^ But, between 2021 and 2022, the study shows a significant decrease in the prevalence. This pattern is consistent with the study in US which showed an accelerated increase from 2017 to 2019 followed by a decline since 2020.^12^ The reason behind the change was said to be a substantial media attention on an increase in adolescent vaping, giving rising to the strict enforcement of the US Food and Drug Administration (FDA) in early 2019 against the sale of e-cigarette cartridges with flavors other than tobacco or menthol and changing minimal age for purchasing e-cigarettes legally from 18 years to 21 years, deterring the adolescent vaping prevalence in US.^12^ In New Zealand, the similar pattern might be explained by the changing vaping policies in New Zealand over time. The New Zealand Government initiated vaping control measures for the first time in 2018, such as prohibiting sales and promotion to those under 18 years, mandating vape-free workplaces, establishing control mechanisms over product safety, like flavours and nicotine concentration,^13,14^ and introducing the ‘Vaping Facts’ website (https://www.vapingfacts.health.nz/) as a trustworthy source about vaping products and their safe use to support stopping smoking. These measures encouraged the use of vaping as a means to quit smoking but may have sent a message to young people and others that vaping was a safe, not ‘safer than smoking’, activity. This delay in introducing controls on vaping, together with a rise in marketing to youth via social media and the advent of new pods and disposable devices, might have contributed to a rapid increase in the prevalence of adolescent vaping in New Zealand between 2014 and 2021. In 2021, the New Zealand Government modified the Smokefree Environments and Regulated Products Act (SERPA) 1990 to focus on regulating retailers, manufacturers, importers, and distributors of vaping products. The Act set timelines for implementing the regulations from 2021 to 2023.^15^ The impacts of this new legislation might partly explain why there appears to have been a decline in the prevalence of adolescent vaping between 2021 and 2022.

One aspect of the SERPA was to make vaping on school grounds illegal.^15^ This might force a punitive approach in school settings that may discourage young people from disclosing their vaping status, but there is no research in New Zealand to support this hypothesis. In addition, there has been no consistent approach by schools – so it is hard to say if any styles of educational approaches have made a difference, or parental interventions. Another explanation for the recent decline could be an effect of the social isolation from the restrictions of the Covid-19 pandemic but there has been mixed evidence on this.^16^ More research will be needed to understand the association of Covid-19 and the related regulations with rates of vaping in New Zealand as well as in other jurisdictions.

Although a one-year drop in the prevalence does not allow any firm conclusions to be drawn, a consistent decline or a slower increase in vaping prevalence over subsequent years might be expected given that the New Zealand Government put consistent efforts and plans in to control vaping during these years; and similar findings (Thailand between 2015 and 2019, Korea between 2011 and 2015, and the US between 2017 and 2019) were identified in our recent scoping review.^17^ Continuous monitoring of the data and vaping regulations could validate the relation between vaping control policies and vaping prevalence and could help make stronger conclusions about New Zealand adolescent vaping conditions, especially for Asian groups.

The prevalence of vaping among Asian adolescents increased at much slower rates compared to that of Non-Asian adolescents. Among Asian major subgroupings, the prevalence of adolescent vaping was higher in the Indian group compared to the other groups in 2022 although the baseline prevalence did not differ much. In addition, unlike other Asian subgroupings’, Indian adolescent vaping increased marginally between 2017 and 2018 and between 2019 and 2021, but then continued increasing between 2021 and 2022. Some factors that might have made Indian adolescents initiate and continue vaping include the ability to create playful tricks with vapour, having fun with friends and siblings, and the influence of social media.^18,19^ Indian adolescents in India have been found to enjoy both uploading social media posts about doing the tricks that they usually learnt from social media and getting ‘likes’ from their social media friends on their posts.^18^ Besides this, they not only used vapes as a group activity with friends and siblings at schools or someone’s home but also easily followed and initiated vaping after friends’ or siblings’ use. In addition, they were found to have a poor awareness of the harms of vaping and perceived them as relatively less harmful than other tobacco products.^18^ Regarding the increase in vaping among adolescents, a collaborative group of Adolescent Medicine Providers from India, Canada, the US and UK suggested public health measures such as targeted public health-led education campaigns, education curricula for schools, and health providers providing messages about the health risks of vaping by adolescents and adults.^20^ Buidling on these recommendations, the New Zealand Government could adopt two culturally appropriate interventions: enhancing parental involvement and initiating peer-led social media campaigns to address the increasing rates of vaping, particularly among Indian adolescents.^19^ Empowering parents through education and support can strengthen their ability to guide and monitor their children’s vaping behaviour. Simultaneously, levaraging social media platforms to deliver relatable and peer-driven messages using culturally relevant language and examples could counter pro-vaping narratives and contents in their digital spaces.^19^

Despite a much lower baseline rate, the prevalence of vaping in Asian adolescent girls increased at a faster rate than in boys and had overtaken them in recent years, like other ethnic groups (2021 and 2022). This finding contrasts with studies from our scoping review which found a stronger association of vaping with male gender.^17^ The years the studies were conducted might contribute to these gender differences because most studies from the scoping view were conducted before 2020, when the prevalence of vaping by boys was still higher than that of girls in New Zealand, according to the ASH Survey findings. However, a 2018 study from California where the higher prevalence of vaping was found in Asian girls identified alcohol consumption as having a strong association with Asian girls’ vaping.^21^ Unfortunately, the ASH survey doesn’t ask questions about alcohol use so we were unable to analyse if any such association was evident in our study. Nevertheless, two possible reasons could account for a higher prevalence of vaping in girls: virtual social factors and the feminine design of many contemporary vaping devices.^22^ Virtual social factors describes exposure to vaping behaviours of friends and influencers on social media.^22,23^ The second reason may lie in recent changes in vape design features that appeal to girls compared to older devices, such as smaller sizes, appealing colours and brand names.^23^ Further research should explore the reasons behind an accelerated increase in vaping prevalence among girls compared to boys in addition to marketing and societal factors that influence their vaping behaviours. Like other ethnic groups, the prevalence of vaping by Asian adolescents who identified as ‘Other gender’ was found to be declining since their baseline period in 2019, but the prevalence throughout the survey-collected years was much higher than that of girls and boys. However, because of the presence of a small number of students identified as ‘Other gender’ in the ASH Survey, a reliable conclusion could not be drawn.

There is mixed evidence around the role that vaping plays in relation to smoking: some analyses suggest that vaping can act as a ‘gateway’ to smoking, whereas there is evidence that vaping may help some young people who smoke to quit.^14^ Also, young people who would have smoked may now choose to vape rather than smoke. In most countries, smoking prevalence is declining across all age groups while vaping is increasing, suggesting that vaping is not a gateway to smoking at a population level.^24,25^ Indeed, the ASH Survey analysis also identified that the prevalence of smoking across all ethnic groups and genders had been consistently declining while the prevalence of vaping was increasing (See Appendix B).

Social supply was found to be a major source of vapes by both Asian and Non-Asian adolescents in New Zealand, a finding consistent with the scoping review finding, which identified peers as the most important contributor to Asian adolescent vaping in both Asian and Western contexts.^17^ However, there was no significant association between social supply and ethnicity in the analysis. This result could be real, or simply due to lack of statistical power to detect any differences rather than the absence of an association. Experimentation was a more important reason for Asian adolescents to vape compared to Non-Asians. This finding is consistent with our scoping review, which also mentioned curiosity as the most common reasons for Asian adolescent vaping in both Western and Asian contexts and a recent systematic review of vaping among Asian Americans which reported curiosity as an increased risk for vaping initation and perceptions and awareness of vapes through their peers or friends as a predictor for lifetime vape use.^17,26^ New Zealand could consider interventions such as peer-led prevention campaigns in school settings that have potential to change beliefs, perceptions, attitudes and behaviours towards health harms and social acceptability of vaping and comprehensive indoor air laws. However, a comprehensive and co-ordinated effort is likely to be needed.^27^

Finally, several findings related to vaping behaviours and attitudes of adolescents in New Zealand are also notable. Pod-based and disposable vapes have become increasingly popular among New Zealand adolescents, probably because of their low price and small size, making them easy to carry and conceal, and convenient to use at any time and place.^4^ Similar findings were found in the US between 2017 and 2018.^4^ In 2022, over 80% of New Zealand adolescents who vaped, including those in Asian groups, used vapes containing nicotine, an increase compared with previous years. Possible reasons behind this change might be the increased availability of nicotine-containing vapes and the arrival of pod devices in New Zealand in 2018.^4^

### Limitations and Strengths

Strengths of our study included its large sample size and the survey’s robust and validated measures, leading to nationally representative coverage of the adolescent population of those ages by Asian ethnic group and subgroupings and gender. Second, we included analysis of adolescents identifying as ‘Other’ gender who are consistently found to have worse health outcomes compared to their counterparts despite their small proportion in the sample. Finally, we included the analysis of time trends in vaping by tracking yearly prevalence of New Zealand Asian adolescent vaping.

Limitations included the cross-sectional design, meaning that we cannot make causal claims about the associations we identified, and the focus on 14 to 15-year-old adolescents who attend schools. It does not reflect the experience of adolescents in other age groups and those who are outside of the formal education system. In addition, sources of vapes and reasons for vaping were grouped according to the options available in the survey so they could not be generalized into other settings. However, individual analysis would also impact the study stastistical power due to data limitation. Nevertheless, experimentation, a key reason for adolescent vaping identified in other studies, was not grouped and analysed separately to improve its generalisability.^17^ Finally, we did not directly compare the Asian group with Māori, Pacific and European adolescents separately.

## Conclusions

In New Zealand, while vaping in Asian adolescents is much less common than in Non-Asian, it has been increasing over time, with some unique patterns within Asian sub-populations. Ongoing monitoring and more in-depth ethnic- and gender-specific research is needed to further understand Asian adolescents' vaping behaviours and so better inform targeted messaging to them, their parents, and schools as well as shaping appropriate national policies and regulations.

## Data Availability

The datasets generated during and/or analysed during the current study are not publicly available due to them being held and released to researchers only on special request by a third party (ASH) but may be made available from ASH on reasonable request.*

## Appendix A: New Zealand Adolescent Data Sources on Tobacco and Vaping

**Table:**

Name of the survey	Frequency of data collection	Measures	Sample size and population	Funder	Remark
Youth Insights Survey (YIS)	Biennial since 2006 From 2012: E-cigarettes	Tobacco proximal risk factors for vaping	Approx 2500 14-15 aged students	Health Promotion Agency	Discontinued in 2018 Little prevalence information
New Zealand Health Survey (NZHS)	Annual since 2011 From 2015/16: E-cigarettes	Tobacco prevalence use only	Approx 14 000 adults with 800-900 15-24 age group	Ministry of Health	Small sample size for 15-17-year-old age so lacking precision
Youth 2000 survey series	Every 5-7 years From 2019: E-cigarettes	Tobacco comprehensive information	Approx 7700 – 10 000 Secondary school students aged 13-17 years	Adolescent Health Research group. Various funders	E-cigarette data was collected for the first time only in 2019 so trend data was not available
ASH Year 10 Snapshot Survey	Annual From 2014: E-cigarettes	Tobacco prevalence and risk factors	Approx 20 000 – 30 000 Year 10 students aged 14-15 years	ASH/Ministry of Health	Limited questions on risk factors

## Appendix B: Prevalence of New Zealand Adolescents Ever and Current Smoking from 2014 to 2022, by Ethnicity, by Gender, and by Asian Major Subgroupings (See Table 1)

The prevalence of ever and current smoking by Asian adolescents decreased - ever smoking from 8.7% in 2014 to 4.5% in 2022 and current smoking from 7.2% in 2014 to 3.8% in 2022. The prevalence of both ever and current smoking by Non-Asian adolescents also decreased, ever smoking from 25% in 2014 to 16% in 2022 and current smoking from 20.2% in 2014 to 11.6% in 2022, respectively. All changes were statistically significant.

The prevalence of ever smoking by Asian girls and Asian boys almost halved between 2014 and 2022, in girls it fell from 7.7% in 2014 to 3.9% in 2022 and in boys from 9.7% in 2014 to 4.7% in 2022. Similarly, the prevalence of current smoking by Asian girls and Asian boys also halved, in girls from 6.3% in 2014 to 3.1% in 2022 and in boys from 8.3% in 2014 to 4.2% in 2022. From 2019 to 2022, the prevalence of ever and current smoking by Asian adolescents who identified as ‘Other’ gender group decreased dramatically, from a very high 42.6% to 15.7% in ever smokers and from 32.8% to 11.4% in current smokers. All the changes in the Asian gender groups were statistically significant.

For Non-Asian adolescents, from 2014 to 2022, the prevalence of ever and current smoking showed a statistically significant decrease, from 26.0% to 16.8% for ever smoking and from 20.4% to 11.7% for current smoking in Non-Asian girls, and from 23.6% to 14.7% for ever smoking and from 19.5% to 11.2% for current smoking in Non-Asian boys. From 2019 to 2022, the prevalence of ever and current smoking by Non-Asians identifying as ‘Other’ gender decreased statistically and significantly, from 39.7% to 20.3% for ever smoking and from 30.9% to 15.8% for current smoking.

From 2014 to 2022, the prevalence of Asian adolescents’ ever and current smoking showed a decrease, from 6.8% to 3.4% for ever smoking and from 6.7% to 3.4% for current smoking in Chinese adolescents, from 9.0% to 4.6% and from 9.0% to 4.5% in ever and current smoking, respectively, in Indian adolescents, and from 9.7% to 5.3% and from 9.6% to 5.3% in ever and current smoking, respectively, in ‘Other Asian’ adolescents. All decreases were statistically significant.
